# Is the Assessment of 5 Meters of Gait with a Single Body-Fixed-Sensor Enough to Recognize Idiopathic Parkinson’s Disease-Associated Gait?

**DOI:** 10.1007/s10439-017-1794-8

**Published:** 2017-01-20

**Authors:** M. E. Micó-Amigo, I. Kingma, G. S. Faber, A. Kunikoshi, J. M. T. van Uem, R. C. van Lummel, W. Maetzler, J. H. van Dieën

**Affiliations:** 1 MOVE Research Institute Amsterdam, Department of Human Movement Sciences, Vrije Universiteit Amsterdam, Amsterdam, The Netherlands; 2 McRoberts B.V., Raamweg 43, 2596 HN The Hague, The Netherlands; 3 Hertie Institute for Clinical Brain Research, Department of Neurodegeneration, Center of Neurology, University of Tübingen, Tübingen, Germany; 4 DZNE, German Center for Neurodegenerative Diseases, Tübingen, Germany

**Keywords:** Short gait episodes, Accelerometers, Gyroscopes, Step-by-step gait analysis

## Abstract

Quantitative assessment of gait in patients with Parkinson’s disease (PD) is an important step in addressing motor symptoms and improving clinical management. Based on the assessment of only 5 meters of gait with a single body-fixed-sensor placed on the lower back, this study presents a method for the identification of step-by-step kinematic parameters in 14 healthy controls and in 28 patients at early-to-moderate stages of idiopathic PD. Differences between groups in step-by-step kinematic parameters were evaluated to understand gait impairments in the PD group. Moreover, a discriminant model between groups was built from a subset of significant and independent parameters and based on a 10-fold cross-validated model. The discriminant model correctly classified a total of 89.5% participants with four kinematic parameters. The sensitivity of the model was 95.8% and the specificity 78.6%. The results indicate that the proposed method permitted to reasonably recognize idiopathic PD-associated gait from 5-m walking assessments. This motivates further investigation on the clinical utility of short episodes of gait assessment with body-fixed-sensors.

**Electronic supplementary material:**

The online version of this article (doi:10.1007/s10439-017-1794-8) contains supplementary material, which is available to authorized users.

## Introduction

Parkinson´s disease (PD) is among the most common neurodegenerative diseases in Europe, with an estimated prevalence of 1.6% in populations above 65 years old. The incidence of PD rises with age and imposes an annual burden on European healthcare systems that approximately ranges between 2600 and 10,000 € per patient. Thus, as the population’s longevity increases, it is expected that PD will impose a growing social and economic burden on societies. Consequently, an increased use of healthcare resources for PD over the following years is expected, which will likely have a significant impact on social security and healthcare systems.^26^

The degeneration of the basal ganglia and the loss of dopaminergic innervations in PD can cause body rigidity, resting tremor and postural instability.^9^ Moreover, they may compromise the speed, automaticity and fluidity or smoothness of movements^4^; reflected in symptoms such as bradykinesia and hypokinesia.^9^ These deficits are frequently present during gait in patients with PD and can be quantitatively and objectively assessed,^4^ providing information regarding the clinical status of the patient. The assessment of gait is part of a widely used clinical rating scale for PD (Unified Parkinson’s disease rating scale, UPDRS)^6^ and is used to monitor and regulate the effects of interventions such as medication, deep-brain-stimulation (DBS) and rehabilitation.^8^ Furthermore, since motor impairments such as trunk rigidity and gait dysfunction are often present at initial stages of the disease, their assessment might lead to earlier and improved diagnosis.^9^ Altogether, the valid assessment of movement in patients with PD is an important step in addressing motor symptoms and improving clinical management.^26^

Altered movement patterns in patients with PD can be detected by analyzing signals recorded with accelerometers and gyroscopes, both integrated in a single device that is located on the lower back.^8^ These signals represent the overall motion pattern given the proximity of the sensor to the center of mass. Their processing enables the assessment of trunk stability, balance control and fall risk.^18^ In addition, the analysis of the recorded signals permits the identification of gait events 11 and the extraction of spatio-temporal gait parameters that are sensitive to patients with motor symptoms of PD.^5^

Accelerometers and gyroscopes, in this context also known as body-fixed-sensors (BFS), are small, light-weight, low-cost sensors with good portability and low-power consumption. Therefore, they are easily applicable and enable the assessment of movement patterns in a clinical setting; potentially enhancing objectivity, sensitivity and reliability of clinical tests.^9^

BFS can be used for the assessment of short episodes of gait, which provide information (gait speed, gait cycle time and stride velocity) that differs from information based on the analysis of long episodes of gait and might be relevant for clinical assessment.^14^ Moreover, these sensors may be easily applicable in view of limited space and time requirements, and given that physical limitations in some patients might be an impediment to performing longer episodes.^11^ Finally, in short gait tests, patients can be challenged to increase their gait speed without causing fatigue. This could have relevant clinical implications, since the performance of gait at high-speed might be different than at convenient speed.^9^,^24^

The relevance for PD of instrumented clinical assessment of short episodes of gait has been supported in several studies.^7^,^10^,^21^ For instance, the gait task evaluation of the Short Physical Performance Battery (SPPB) protocol has been reported to be related to disability and PD severity.^21^ In addition, spatio-temporal parameters derived from low-back accelerometry of 10-m walking assessment resulted sensitive to dopamine agonist treatment.^7^ The assessment of short episodes of gait with BFS is also well suited to study gait initiation, which can be affected in patients with PD.^10^ It is therefore conceivable that acceleration, steady-state and deceleration phases of gait episodes are all informative in the assessment of PD-related motor impairments.

To our knowledge, there are no published studies reporting a phase-by-phase analysis of short episodes of gait in patients with PD. Therefore, in this study, episodes of 5-m gait were assessed with a single BFS placed on the lower back that integrated a triaxial accelerometer and a triaxial gyroscope. This permitted to calculate step-by-step kinematic parameters in patients with PD at early-to-moderate stages and healthy control (HC) subjects. Significantly different parameters between groups were evaluated to understand gait impairments in the PD group (PDg). Moreover, from a selection of these parameters, a discriminant model between groups was built. With this, we aim to recognize idiopathic PD-associated gait from low-back accelerometry data of 5-m walking assessments.

## Materials and Methods

### Subjects

This cross-sectional study was performed with 38 participants, 24 patients diagnosed with idiopathic PD and 14 healthy controls, well matched for age and gender (see Table 1). All participants were recruited from the clinical ward and the outpatient clinic of the Neurodegenerative Department from University Hospital of Tübingen, Germany.

The Declaration of Helsinki was respected, local ethical committee approval was obtained (Tübingen 140622) and all subjects provided informed written consent for participation in the study and for publication of individual, anonymized data.

The participants were selected according to the following inclusion criteria: (a) age between 40 and 85 years; (b) ability to walk 10 meters independently and stand safely without walking aid; (c) absence of any psychiatric problem; (d) absence of dyskinesia. All participants underwent a clinical assessment which included: medical history, medication intake and neurological examination.

The participants of the PDg were diagnosed with idiopathic PD according to the United Kingdom Brain Bank Society criteria, in stage 1 to 2.5 (medication ON) of the Hoehn & Yahr scale and with a minimum score of 25 points on the Mini Mental State Examination score. They did not present any other neurological disease. Six subjects with PD (25%) had undergone a DBS operation. All the patients were recruited in their regular ON medication state. The medication ON condition was defined as a time period of 30 min to 3 h after the intake of the usual dose of dopaminergic medication and considering each participant’s perception of having a “Good On Phase”. The participants of the control group had no neurological disease and no relevant intellectual deficits.

An overview of demographic and clinical data is presented in Table 1.

**Table 1:** Demographic and clinical data presented as mean ± standard deviation for the parametric data and median [range] for the non-parametric data, marked with *. In the case of gender, the data is presented as a number of females and (the percentage of females, over the total number of participants for each group). Hoehn & Yahr score (H&Y); Mini Mental State Score (MMSE).

### Protocol

All subjects, wearing their own shoes, walked a 5 meters long track after an acoustic start signal. The track was demarcated by four templates of adult-sized footprints, two attached to the floor at the start position and two at the end position of the track. At the beginning of each trial the subjects stood over the start footprints for at least 3 s. After an acoustic signal, the subjects started to walk the 5-m track. The trial ended when the subjects reached the end, placing their shoes on the footprint templates. Afterwards, the subjects stood still for 3 s before repeating the trial. Two trials were performed at self-selected gait speed (SS) and one trial as fast as possible (FS).

### Instrumentation

The system consisted of a BFS (DynaPort^®^ Hybrid, McRoberts), a remote control and a portable computer on which the DynaPort McRoberts software was installed. The three were synchronized via Bluetooth. The sensor includes a triaxial accelerometer and a triaxial gyroscope, storing data at a sampling rate of 100 samples/s. The accelerometer has a resolution of 1 mg (0.0981 m/s^2^) and is a DC type sensor, sensitive to gravity.

The sensor was inserted in an elastic belt, placed around the waist so that the sensor was positioned at the level of the lowest lumbar vertebra (L5). Then, the software was activated and this initiated data collection. At the beginning and end of each trial an acoustic signal was manually triggered with the remote control and stored.

In addition, two BFS (DynaPort MiniMod McRoberts) were attached to the lateral sides of both heels in 22 patients with PD. These BFS include a DC type triaxial accelerometer with a sample rate of 100 samples/s and a resolution of 1 mg (0.0981 m/s^2^).

Data acquisition was synchronized for all the collected signals using an impulse that was transferred to each of the systems.

### Calculation of Kinematic Parameters

**Figure 1:**
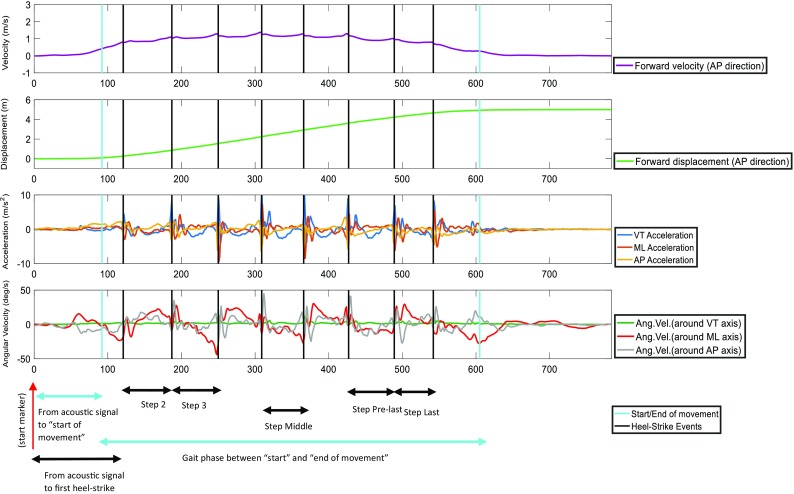
Typical example of forward velocity signal (in AP direction), forward displacement signal, acceleration signals in the three axes and angular velocity signals in the three planes from a healthy control subject.

**Figure 2:**
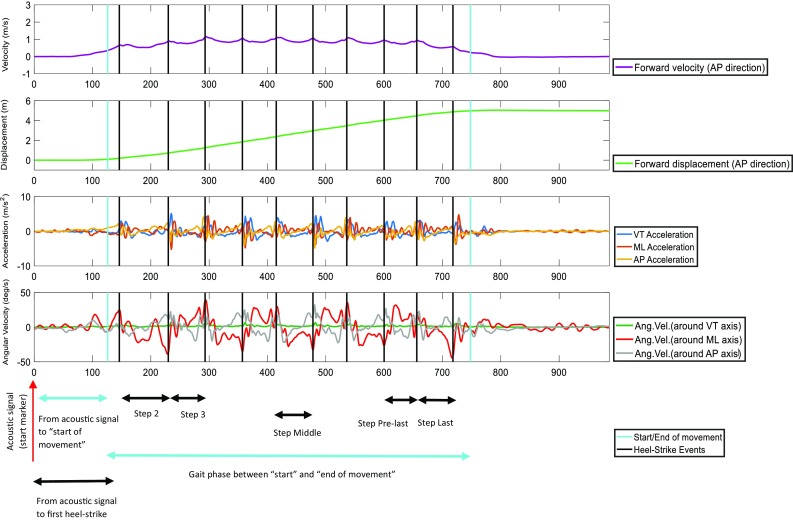
Typical example of forward velocity signal (in AP direction), forward displacement signal, acceleration signals in the three axes and angular velocity signals in the three planes from a subject with Parkinson’s disease.

Velocity and displacement (Figs. 1, 2) in the anterior-posterior direction (AP) were calculated from the raw acceleration and angular velocity signals recorded on the low-back, including the pre and post standing phases.^27^ Further details of these calculations are found in the Appendix 1 of supplementary material.

#### Phases

The “start of movement” was defined as the first instant at which the AP velocity exceeded 30% of the maximal AP velocity; whereas the “end of movement” was defined as the last instant at which the AP velocity exceeded 20% of the maximal AP velocity (for the SS condition) and 15% of the maximal AP velocity (for the FS condition). The low-back acceleration in the vertical direction (VT) was low-pass filtered with a bi-directional fourth order filter and a cut-off frequency of 3.5 Hz. Subsequently, the first heel-strike was defined as the instant of the first peak after the “start of movement”. With the aim to automatically segment the signal delimited by the “start” and “end of movement” in step cycles, a template-match method 11 was applied to the low-back raw AP acceleration signal. Further details of this method are found in the Appendix 2 of supplementary material.

The performance of the algorithm was tested over data from 22 subjects of the PDg. Likewise calculated in previous work, this was tested by comparing absolute average differences within trials and across subjects in stride duration between estimates obtained from the application of the algorithm to low-back accelerometry and estimates derived from heel accelerometry (see Appendix 2 of supplementary material).

The following gait phases were determined:

1. Gait phase delimited between the “start” and the “end of movement”.
2. From the acoustic signal to the “start of movement”.
3. From the acoustic signal to the first heel-strike.
4. Second step.
5. Third step.
6. Middle step.
7. Pre-last step.
8. Last step.

#### Signal Features

Between the “start” and “end of movement”, the average gait speed of the trial was calculated as the ratio of displacement and duration. In addition, the number of steps was counted. The corrected displacement, angular velocity and acceleration signals were used to calculate kinematic parameters (Figs. 1, 2).

For all the gait phases, the following features were calculated:

- Duration.
- Forward displacement (AP).
- Range of forward velocity (AP).
- Root-mean-squared (RMS) value (variability around the mean) of Acceleration VT.
- RMS of Acceleration ML (medio-lateral direction).
- RMS of Acceleration AP.
- RMS of Angular velocity around VT axis.
- RMS of Angular velocity around ML axis.
- RMS of Angular velocity around AP axis.

Furthermore, the mean values across steps were calculated for all the parameters. Subsequently, kinematic parameters for each of the gait phases were calculated relative to the mean value across steps (denoted as “relative”). As a result, for both conditions (SS and FS), 160 kinematic parameters were obtained (Tables 2, 3).

**Table 2:** Results of kinematic parameters for the self-selected gait speed condition (SS). The top number in each cell is the mean percentage of differences, calculated as the mean value for outcomes from PDg minus mean value for outcomes from the HCg, relative to the mean value for outcomes from the HCg. The bottom number is the corresponding *p* value for the difference (all parameters with *p* < 0.05 are marked with a grey background). Note that the results corresponding to kinematic parameters relative to the mean value across steps are marked with *R.

**Table 3:** Results of kinematic parameters for the fast gait speed condition (FS). The top number in each cell is the mean percentage of differences, calculated as the mean value for outcomes from PDg minus mean value for outcomes from the HCg, relative to the mean value for outcomes from the HCg. The bottom number is the corresponding *p* value for the difference (all parameters with *p* < 0.05 are marked with a grey background). Note that the results corresponding to kinematic parameters relative to the mean value across steps are marked with *R.

All calculations were performed with a custom Matlab program (version 7.10.0. Natwick, Massachusetts: The MathWorks Inc., R2010b).

### Statistical Analysis

The Shapiro–Wilk test (for platykurtic samples) and Shapiro–Francia test (for leptokurtic samples) were implemented in Matlab to test normality of data distribution. Accordingly, unpaired t-tests and Wilcoxon Rank tests were used to assess differences between groups for each of the calculated kinematic parameter. For the SS condition, parameters obtained from both gait trials were averaged per subject. Significance level was set at *α* = 0.05 for all analyses. We did not correct the *p* values for multiple comparisons, as this is an explorative study and we were concerned about possible Type II errors. However, instead of selecting some of the results with specific *p* values, we avoided any *p* hacking and reported all significant as well as non-significant results to allow interpretation based on the pattern of results.

The percentage of difference in average parameters between both groups was calculated relative to the average in the HC group (HCg). For the significantly different parameters, sensitivity and specificity were calculated by choosing the best classification threshold. In addition, the harmonic mean of sensitivity and specificity (F1) was calculated.

A stepwise discriminant analysis was separately performed for the outcomes of the SS condition and the outcomes of the FS condition, using Statistical Package for the Social Sciences (SPSS), version 22. First, significantly different parameters between groups were preselected. Then, the correlations between these were calculated (see Appendix 3 of supplementary material). Next, from the preselected parameters, the non-normally distributed were excluded. Moreover, in case of absolute correlations above 0.7 between parameters, the one with the highest *p* value in the test for difference between groups was also excluded. Subsequently, the remaining parameters (see Appendix 3 of supplementary material) were inserted in a forward stepwise discriminant analysis. The sensitivity and specificity of the model were additionally calculated by a 10-fold cross-validated discriminant analysis.

## Results

Based on visual inspection, the proposed algorithm detected all the steps/strides without false positives and without false negatives when applied respectively on both low-back accelerometry and heel accelerometry. Absolute average differences in stride duration within trials and across subjects between methods were on average 31.1 ± 5.4 ms (5.4 ± 2.4% of average step duration and ICC = 0.87).

For the phase delimited between the “start” and “end of movement”, there were no significant differences between groups in number of steps (*p* = 0.95 for SS condition, *p* = 0.17 for FS condition), nor in average gait speed (*p* = 0.42 for both, SS and FS condition). Table 2 (SS condition) and Table 3 (FS condition) present, for all kinematic parameters, the percentage difference between averaged values of both groups and the *p* values of tests for differences between groups. Further details regarding the correlations between all significantly different parameters between groups, and the mean and standard deviation of all parameters can be found in Appendices 3 and 4, respectively.

In both conditions, most of the significantly different kinematic parameters between groups were found at initial gait phases (17 out of 31 and 5 out of 16 significant differences for the SS and FS condition, respectively). Furthermore, in the SS condition, most of the significant differences were found for kinematic parameters expressed relative to the mean across steps (19 out of 31 significant differences). For both conditions, the largest number of kinematic parameters that were significantly different between groups was found for duration (6 gait phases in the SS condition and 4 gait phases in the FS condition) and RMS of angular velocity around the AP axis (7 gait phases in the SS and 4 gait phases in the FS condition). In the case of FS condition, also 4 gait phases were significantly different between groups for the RMS of angular velocity around the ML axis. Notice that some of the mentioned parameters were not independent from each other (see Appendix 3 of supplementary material).

Percentages difference between groups under the SS condition show that the PDg required a longer time than the HCg to initiate the movement. However, the middle step, pre-last step and mean duration across steps were shorter in the PDg. In the FS condition, a comparable reduced duration at intermediate and final steps was found for the PDg, whereas no differences with the HCg were found at gait initiation.

**Figure 3:**
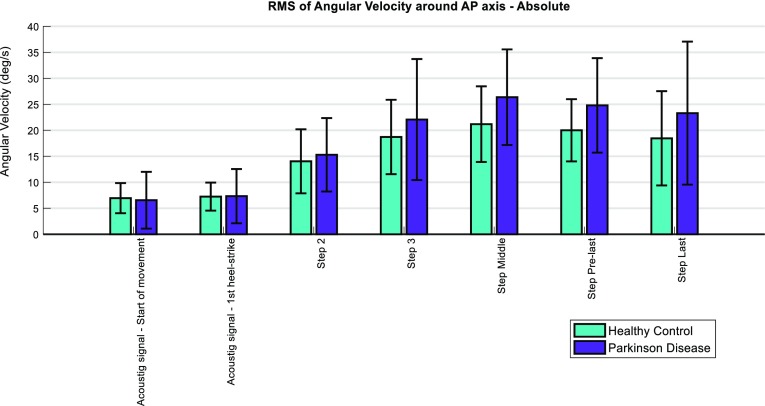
Absolute values of RMS of Angular Velocity around AP axis for each gait phase. The error bars correspond to the standard deviation of the outcomes from each group.

**Figure 4:**
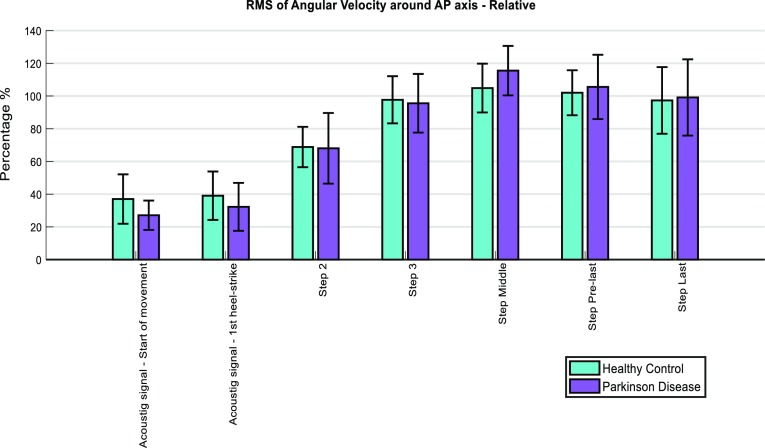
Values of RMS of Angular Velocity around AP axis for each gait phase, relative to the mean value across steps. The error bars correspond to the standard deviation of the outcomes from each group.

In both conditions (SS and FS), the PDg had an increased sway around the AP axis (relative to the HCg) at the phase delimited between the “start” and “end of movement” and at the middle step. This was evidenced by a higher RMS of angular velocity around the AP axis (Fig. 3; Tables 3 and 4 from Appendix 4 of supplementary material). Conversely, relative values of this feature at initial gait phases were lower in the PDg than in the HCg (Fig. 4).

F1 scores ranged between 0.63 and 0.69 for parameters of SS condition, and between 0.64 and 0.66 for parameters of FS condition. The highest F1 score (0.69) in the SS condition was found for the relative RMS of angular velocity around the AP axis at the start of movement (between the acoustic signal and the “start of movement”, and expressed relative to the mean across steps). The highest F1 score (0.66) in the FS condition was found for the duration of the middle step.

From the stepwise discriminant analysis applied with variables calculated from the SS condition, we obtained 4 predictors: relative RMS of VT acceleration at the second step, relative displacement at the start of movement, range of AP velocity at the pre-last step and relative RMS of VT acceleration at the pre-last step. The discriminant function significantly differentiated the groups (Λ = 0.39, 
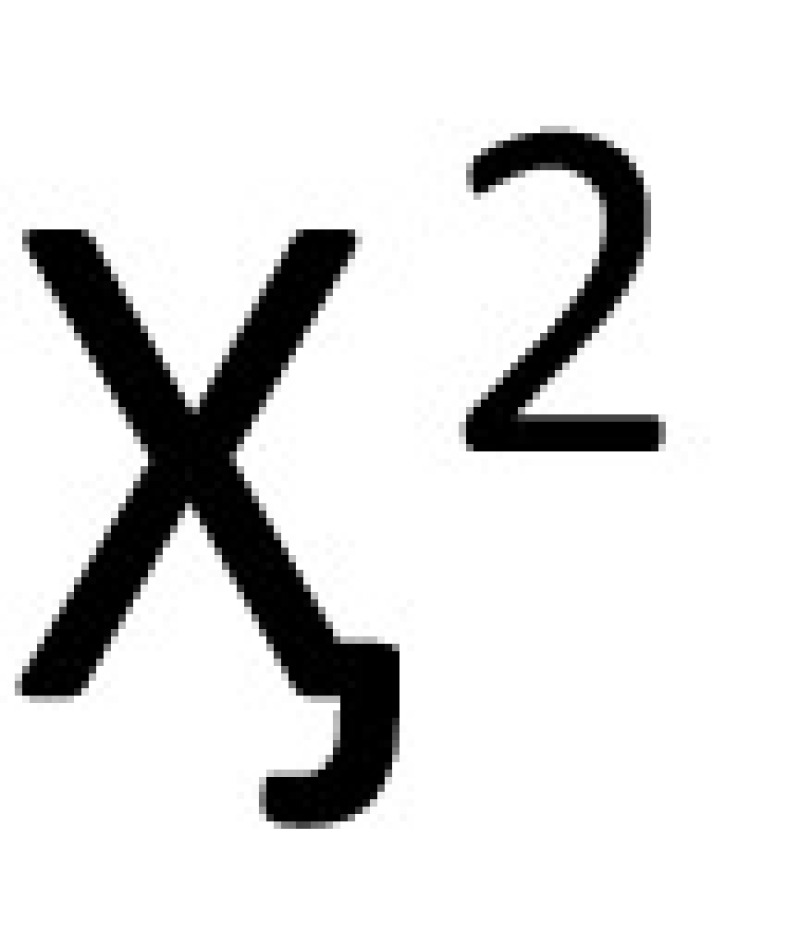
 (2) = 32.09, *p* < 0.001). A total of 92.1% of participants were correctly classified; the sensitivity of the predictive model was 100% and the specificity 78.6%. With the 10-fold cross-validated discriminant analysis, 89.5% participants were correctly classified; the sensitivity decreased to 95.8% and the specificity remained at 78.6%.

From the stepwise discriminant analysis applied with variables calculated from the FS condition, we obtained 2 predictors: duration of the middle step and RMS of angular velocity around ML axis of the last step. The discriminant function significantly differentiated the groups (Λ = 0.61, 
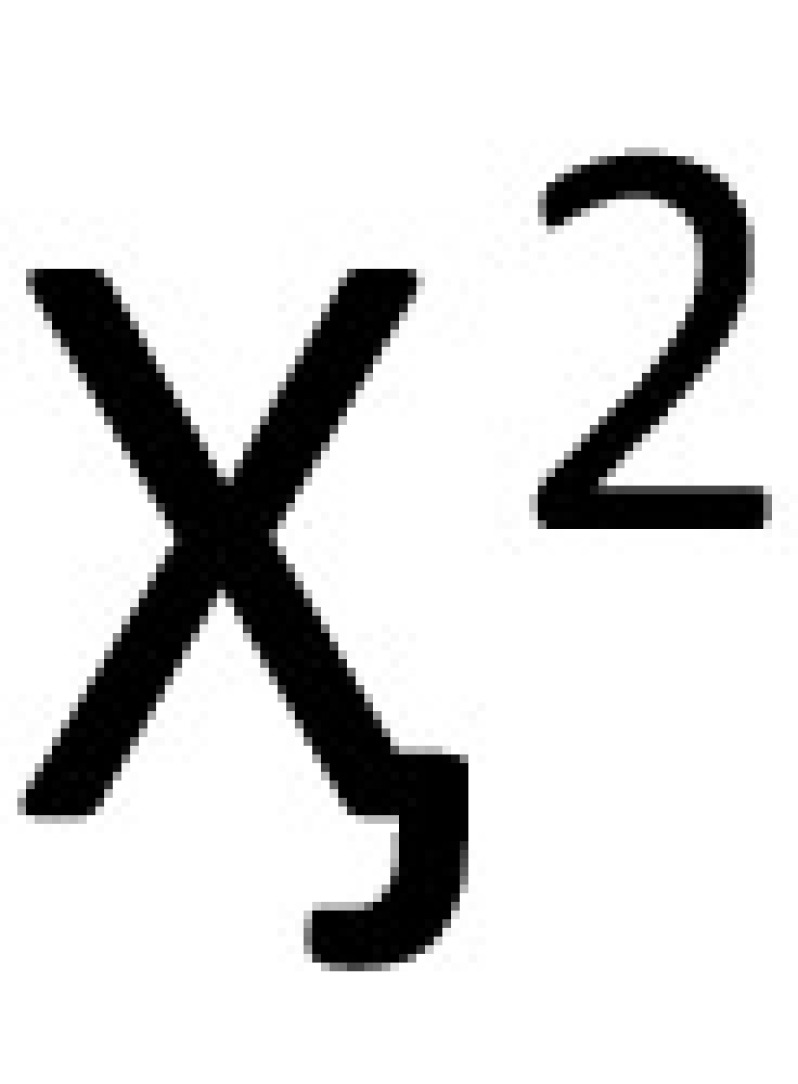
 (2) = 16.78, *p* < 0.001). A total of 81.6% of original grouped cases were correctly classified; the sensitivity of the predictive model was 87.5% and the specificity 71.4%. The same results were found from the 10-fold cross-validated discriminant analysis.

## Discussion

Based on the assessment of only 5 meters of gait with a single BFS placed on the lower back, this study presents a method for the identification of step-by-step kinematic parameters in HCs and in patients at early-to-moderate stages of idiopathic PD. Significant differences between groups were found for certain parameters, enhancing the understanding of gait impairments in PD. Moreover, a selected number of these parameters permitted to classify idiopathic PD-associated gait with reasonable precision.

### Gait Segmentation

The proposed algorithm segmented gait episodes in step cycles with lower accuracy (*p* < 0.01) for the PDg than for previously reported results from a HC cohort (with differences of 3.4% in average step duration).^11^ This is possibly due to an asymmetrical and variable gait and maybe due to pathological step initiation, common in patients with idiopathic PD.^3^,^10^ We have performed a post-hoc analysis, comparing all the estimates when excluding the first event and we obtained a lower (*p* = 0.01) absolute average difference: 21.1 ± 12.6 ms (3.7 ± 2.2% of average step duration and ICC = 0.93). This indicates a lower accuracy on the detection of the first event, possibly due to the different nature of the acceleration signals along the first step with respect to the other steps.

The obtained results were comparable to reported^22^ differences in step cycles between estimates from accelerometry and gold standard methods in a PDg. This implies that this method is also appropriate for the analysis of gait in patients with idiopathic PD at early-to-moderate stages.

### Gait Initiation

Several studies^3^,^10^ have shown that patients with PD often present impairments in anticipatory postural adjustments and consequent difficulties in step initiation, suggesting that the calculation of parameters over initial gait phases is of special clinical interest in the study of gait in PD. Indeed, most of the significantly different parameters between groups of this study were derived from initial gait phases. Particularly, under SS condition, the PDg initiated stepping with longer duration than the HCg. This, in agreement with other studies, showed that movement latency was longer^12^ and step preparation was slower^2^,^20^ in the PDg. Prolonged duration at initial gait phases in the PDg imply delays in reaction time and/or delays in gait initiation. These delays might also contribute to lower RMS of acceleration and angular velocity signals, since longer static periods prior to gait initiation lead to lower RMS values over these intervals. These findings are also consistent with larger displacement at the time that 30% of maximal AP velocity is reached, which might reflect in a more pronounced bending from the trunk in the PDg. Combined, these results suggest that decreased values of signal fluctuations at initial gait phases are not only related to delays in movement initiation, but also to changes in kinematics.

Lower values for the PDg in RMS of acceleration and angular velocity signals at gait initiation, relative to the mean across steps, might also reflect impairments in step preparation^2^,^20^ and impaired anticipatory postural adjustments,^12^ particularly in the SS condition. These problems might be due to a primary balance deficit: the inability to properly shift weight to one leg, normally required for contralateral limb swing.^3^ Note however that relative rather than absolute RMS values of acceleration and angular velocities were affected, which might indicate that an increased sway post-initiation of gait, rather than reduced sway during initiation itself, may contribute to these differences. We speculate that these findings indicate a more stiff movement in the PDg than in the HCg, possibly due to reduced intersegmental articulation,^19^ increased axial rigidity^23^ and impaired control over pelvis rotation.^25^ Earlier work^23^ observed in-phase movements between pelvis and thorax in patients with PD at self-selected speed, whereas out-of-phase movements were observed in control subjects; suggesting that the assessed PDg had different trunk coordination from the HCg.

### Post-initiation of Gait

Shortened step duration is often seen in patients with PD,^20^ possibly as a consequence of trunk rigidity^23^ or reduced lower-extremity extension-flexion movements.^19^ Accordingly, intermediate, pre-last steps and mean values across steps were of shorter duration in the PDg under both conditions (SS and FS). However, step length was not as clearly affected as step duration. This could be related to errors in the calculation of step displacement inherent to the integration drift. Nevertheless, under the SS condition, the displacement of the second step relative to the mean across steps was significantly reduced for the PDg. This indicates that while the displacement right after gait initiation seems not to be affected, increases in displacement from the second step to the rest of steps were more pronounced for individuals with PD than for HCs.

The regulation of stride length, not the regulation of step timing, is considered the central motor disruption in gait hypokinesia and requires an increase in cadence as a compensatory mechanism.^13^ This was not present in our results as the number of steps within the phase delimited by the “start” and “end of movement” was not significantly different between groups. Lack of significant differences between groups in number of steps, displacement, duration and average gait speed calculated for the phase between the “start” and “end of movement” might be due to the lack of consistency in the duration of this phase across subjects of the same group. Another potential reason for this could have been the exclusion of the complete acceleration and deceleration gait phases for the calculation of the mentioned features. Especially the acceleration phase took longer and, more importantly, the distance covered before the “start of movement” was 50% larger in patients with PD. Differences may also have gone unnoticed due to the short length of the walking track. It has been shown^14^ that older adults change their walking strategy as a function of walking distance. This suggests that not only the PDg, but also the HCg could have performed gait over 5 meters with different strategies than in most of the studies based on longer, and thus more steady gait protocols.

The RMS of angular velocity around the AP axis is related to lateral sway and was sensitive to differences between groups, not only as a mean value across steps, but also at intermediate gait phases. Larger values of this feature in the PDg at intermediate gait phases suggest that once the propulsion in locomotion is achieved, the frontal plane rotational velocity in subjects with PD fluctuates more than in HCs, which might reflect difficulties in the PDg to laterally control the movement.^20^

### Deceleration Phase in Gait

The assessment of the final gait phases is clinically interesting since the ability to stop forward progression of the body’s center of mass^15^ and the maintenance of balance^16^ are challenged at gait termination. Moreover, patients with PD perform decelerating gait phases with altered strategies.^15^ This is reflected in our study at the pre-last and last steps prior to the drop of AP velocity. In the SS condition, individuals with PD performed the pre-last step with decreased range of AP velocity; suggesting that this group may have prepared the end of locomotion by reducing movement intensity differently from the HCg. Furthermore, larger fluctuations in the PDg for the angular velocity around AP axis (SS condition) and around ML axis (FS condition) along the last two steps indicate difficulties in patients with PD to maintain balance while stopping. Additionally, larger values of the displacement at the last step relative to the mean across steps were obtained for the PDg. This indicates that the PDg covered longer distances per step (than the HCg) towards the end of the gait trial.

### Sensitivity of Kinematic Parameters

Most of the significantly different parameters between groups and the lowest *p* values were obtained for relative values with respect to the mean across steps, being most of them independent from each other. This suggests that the segmentation in step cycles of short episodes of gait and the extraction of features within these phases, additionally to the extraction of mean features across steps, permits the identification of PD sensitive parameters.

All the kinematic parameters that differed between HC and PD subjects had a similar classification power, indicated by similar F1 scores. However, from the results of the discriminant analysis we observe that the combination of several parameters considerably (about 20%) increases the classification power of the model. This is comparable to the classification power of methods based on the assessment of 10-m gait with feet accelerometry.^1^

In the final statistical models, half of the predictors were based on RMS values, which suggests that fluctuations of acceleration and angular velocity signals are appropriate features to assess from short episodes of gait for the recognition of idiopathic PD-associated gait. The remaining predictors for the SS model (relative displacement at the start of movement and range of AP velocity at the pre-last step) and for the FS model (duration of the last step) indicate that spatio-temporal information form initiation and ending of gait is also relevant and complementary to discriminate PD patterns from HCs.

Previous studies have shown that gait and balance functions are relatively well conserved in self-selected (or convenient) assessment conditions,^17^ whereas subtle deficits are revealed in subjects with PD under more challenging conditions.^9^,^24^ Conversely, our findings indicate that the analysis with the proposed method of short episodes of gait in SS condition permits a better recognition of idiopathic PD-associated gait (from patients at early-to-moderate stages) than in FS condition. This is clinically relevant, since the assessment of gait in SS condition is more feasible than in the FS condition when physical limitations in some patients impede performance of fast gait and/or this becomes a burden for the participants. Note that in the present study only one trial was performed for the FS condition (to limit the burden for the participants) against two trials for the SS condition, which may have affected the discriminant power of the FS condition.

### Applicability and Usefulness of this Method

Impairment in ambulation and lower-limb motor planning are the main determinants of falls in patients with PD. Particularly, deficits in anticipatory postural adjustments cause gait akinesia and could lead to difficulties initiating a compensatory step, which is crucial in balance correcting strategies to prevent patients from falling.^2^ In this regard, the analysis of initial gait phases, as proposed in this study, may be relevant in the evaluation of risk of falling and the effect of dopaminergic therapies.^3^ On the other hand, the analysis of spatio-temporal outcomes extracted from short episodes of gait such as gait speed, step timing and step length might be useful as early markers of the disease; reflecting the decline of motor automaticity and bilateral motor control of gait.^28^

The use of BFS in the assessment of such short episodes of gait adds a significant value compared to the use of stopwatch measurements such as total duration or gait speed. It permits to include step-by-step analysis of several features which are sensitive to PD and whose calculation does not rely on manual marking.

Altogether, the quantitative assessment of short (5-m) walking distances with this novel method may contribute to the understanding of gait impairments in a clinical setting. However, future work should compare the proposed outcomes to more common clinical assessments, such as the UPDRS and the SPPB, in order to test the clinical value of the proposed technique.

### Limitations

Several limitations of this study must be mentioned. First, our findings cannot be generalized to the overall PD population, since patients with PD and relevant cognitive impairment were excluded. However, the focus of this study was on the assessment of parameters related to gait motor symptoms of patients at early-to-moderate stages of idiopathic PD. Second, while the main objective of this study was to explore differences in features of short gait episodes between groups in order to recognize PD-associated gait, the reliability and robustness of the parameters still need to be tested. Third, short episodes of gait lack of steady-state phase and present a limited number of cycles. Therefore, kinematic parameters of important clinical value such as gait variability, gait symmetry and local dynamic stability are neither valid, nor reliable when estimated from such data. Fourth, step cycles were delimited by events that were defined as the instants of maximal matching between the template and the signal. Thus, the segmentation in step cycles based on a template-matching algorithm depends on the selection of a specific template (obtained as an average of gait cycles from an individual gait trial) and the selected gait event can slightly vary between subjects. But using the same criteria for each of the subjects, these events approximately correspond to heel-strike events and the periodicity of step cycles is obtained with acceptable accuracy. On the other hand, while a gold standard was not available, the accuracy of step detection was tested by comparing estimates from heel accelerometry. This was considered adequate for the purpose because of the magnitude of the heel-strike peaks in heel acceleration signals and the proximity of the sensor to the location where the ground reaction force impacts.^11^ Additionally, based on this method, low accuracy in the detection of the first and the last steps was expected. Consequently, the first heel-strike was separately calculated to define the phase between the acoustic signal and the first heel-strike. Moreover, the last heel-strike was not included in the analysis, since the “end of movement” was defined at the last drop of velocity, prior to the last and positioning step. Finally, the sample size of this cross-sectional study (with relative lower size for the HCg than for the PDg) might have resulted in limited statistical power. Thus, this study should be considered as an explorative study, albeit that previous publications related to gait assessment in PD used comparable group size populations.^1^,^5^

## Electronic supplementary material

Below is the link to the electronic supplementary material.

Supplementary material 1 (PDF 1067 kb)

## Abbreviations

PD: Parkinson’s disease
HC: Healthy control
DBS: Deep-brain-stimulation
BFS: Body-fixed-sensors
SS: Self-selected speed
FS: Fast speed
VT: Vertical direction
ML: Medio-lateral direction
AP: Anterior-posterior direction
