# Supplementary material for: Is the Assessment of 5 Meters of Gait with a Single Body-Fixed-Sensor Enough to Recognize Idiopathic Parkinson’s Disease-Associated Gait?
Source: Ann Biomed Eng. 2017 Jan 20;45(5):1266–78. doi: 10.1007/s10439-017-1794-8 (PMC5397518; doi:10.1007/s10439-017-1794-8)
Supplement: Supplementary file 1 — Supplementary material 1 (PDF 1067 kb) [file 10439_2017_1794_MOESM1_ESM.pdf]

Appendix 1

Velocity and displacement in the AP direction were calculated from the raw acceleration and angular velocity signals recorded on the low-back, including the pre and post standing phases, using the following calculation steps:

1. During the stationary initial and final standing phases, the sensor orientation about the global horizontal axes (sensor inclination/tilt angle) was calculated based on the accelerometer's reading of gravity (when an accelerometer is held still, it only measures the gravitational acceleration)[1]. The heading angle of the sensor (angle about the global vertical) was assumed to be zero during the stationary phases.

2. From this start orientation, the angular velocities were integrated to obtain the sensor orientation over time.

3. Due to integration drift, the sensor orientation at the end of the trial slightly differed from the end position that was calculated based on the acceleration signals (step 1). This accumulated integration error was used to correct the sensor orientation time series that were calculated in step 2. To get a smooth curve, the error correction was distributed over the whole movement period (rotating about the helical axis of the error matrix with respect to the identity matrix), assuming a linear error increase over time[3].

4. Using this corrected sensor orientation, the locally measured sensor accelerations and angular velocities were rotated to the global coordinate system.

5. Because the subjects were standing still at the start and end of the trial, the average global acceleration should be zero in all directions. Therefore, the mean value was subtracted from each acceleration signal (thereby also removing the offset in the vertical direction due to the effect of gravity).

6. The corrected accelerations were then integrated and double-integrated to obtain the velocity and position, respectively.

7. Because the sensor was not perfectly aligned with the trunk segment and the subjects were never facing the target, the AP axis of the sensor was not pointing forward during the initial/final standing phases. This resulted in a calculated significant sideward displacement between the start and end position. To correct this, positions, velocities and accelerations were rotated around the vertical axis, such that the sideways displacement between the start and end standing phases was zero.

8. Due to the integration drift, the calculated walking distance at the end of the trial differed slightly from the 5 m walking distance. To correct this, both position and velocity were scaled by the ratio between the actual 5-m walking distance and the estimated walking distance at step 6.

Appendix 2

Algorithm for gait segmentation in step cycles from low-back accelerometry

The algorithm is based on the acceleration in the AP direction. Firstly, it defines a template, which represents a typical pattern of step cycle acceleration and subsequently it searches for the periods of maximal match between the signal and the template. The raw acceleration signal obtained from the accelerometer was delimited by the start and the end of movement and denoted as "Segmented signal". Based on low-back accelerometry and following the flowchart (figure 1), the algorithm executes the subsequent operations:

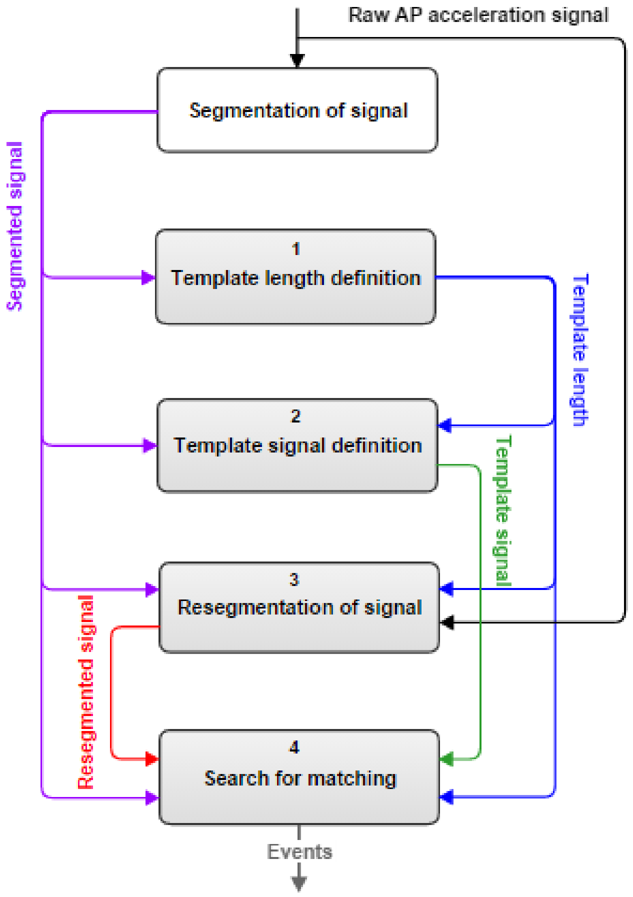

Figure 1. Flowchart that represents the operations executed by the algorithm to segment the signal in step/stride cycles.

1. Template length definition

In this block the length of the template signal was defined using the “Segmented signal” as an input and following these steps:

1.1. Obtain the unbiased auto-covariance signal of the input with the function < xcov, unbiased > from Matlab Signal Processing Toolbox 7.11.0.

1.2. Extract the dominant frequency from the unbiased auto-covariance signal (positive lags).

1.3. Calculate the inverse of the dominant frequency and multiply it by the sampling frequency. The Template Length (TL) is defined as the resulting number of samples.

2. Template signal definition

In this block the “Template signal” is defined, using TL and the “Segmented signal” as inputs.

2.1. Define a low limit on the “Segmented signal” after 115% TL samples from the start, and a high limit 115% TL samples before the end.

2.2. Find peaks in the “Segmented signal” between the low and high limits, which are at least 40% TL samples from each other.

2.3. Define sections of the input signal around each peak, starting 15% TL samples before the instant at which the peak is found (with the aim to include the slope preceding the peak), and ending 100% TL samples after the instant at which the peak is found.

2.4. Obtain new signals from the application of the dynamic-time-warping technique (DTW)[2] on each of the sections and their consecutive ones. This technique optimally aligns the sections, combining them in an average signal.

2.5. Repeat step 2.4 until a single new signal, named “Template signal”, is obtained. The length of this signal is TL samples. This signal is the average of all the sections defined in step 2.3 (figure 2.a).

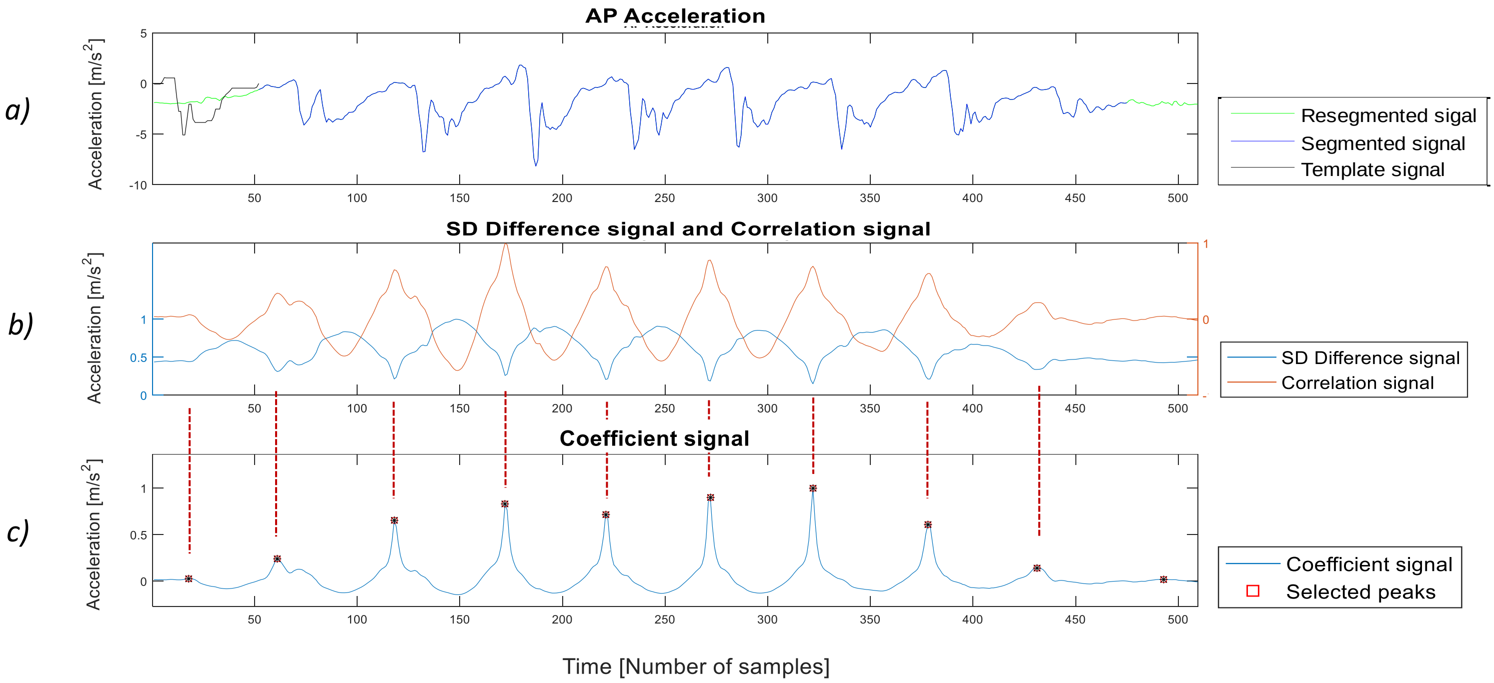

Figure 2. a. Typical example of a “Segmented signal” (blue), a “Resegmented signal” (green) and a “Template signal” (black), obtained as average of all the sections defined in step 2.3.

b. Typical example of a “SD Difference signal” (blue) and a “Correlation signal” (green). The “SD Difference signal” was obtained from the standard deviation of the difference in amplitude between the “Template signal” and a sliding window through the “Resegmented signal”. “Correlation signal” was obtained from the calculation of correlation coefficients between the “Template signal” and a sliding window through the “Resegmented signal”, being multiplied by the ratio of ranges of the “Resegmented signal” and the “Template signal”.

c. “Coefficient signal” (blue) and “Selected peaks” (red squares).The normalized ratio signal between the “Correlation signal” and the “SD Difference signal” permitted to obtain the “Coefficient signal”. “Selected peaks” are the peaks from the “Coefficient signal” which are found within the dimension of the “Segmented signal” and are located at a distance of at least a 60% TL (template length) samples from each other.

3. Resegmentation of signal

In this block a new segmentation of the raw acceleration signal is performed using TL, the raw acceleration signal, and the start and end sample number of the “Segmented signal” as inputs.

3.1. Extend the segmentation of the “Segmented signal” to the left with TL samples, and to the right with twice TL samples from the original raw acceleration signal. The resulting signal is denoted as “Resegmented signal”, the output of this block.

4. Search for match between “Template signal” and “Resegmented signal”

In this block the match between the “Template signal” and the “Resegmented signal” is found. The aim is to extract the periods from the “Resegmented signal” in which the acceleration resembles the template in

magnitude and shape. This permits to evaluate the periodicity of steps. The inputs of this block are template length (TL), the “Template signal”, the ¨Segmented signal” and the “Resegmented signal”.

4.1. Calculate a signal based on the standard deviation of the difference in amplitude between the “Template signal” and a sliding window (with TL samples) through the “Resegmented signal”.

4.2. Normalize the resulting signal. This signal, denoted as “SD Difference signal”, has local minima at the start of the intervals along which the “Resegmented signal” and the “Template signal” have the best match, and therefore are more similar in shape and amplitude (figure 2.b).

4.3. Calculate a signal based on the calculation of correlation coefficients (using the function < corrcoef > from Matlab Signal Processing Toolbox 7.11.0) between the “Template signal” and a sliding window through the “Resegmented signal”.

4.4. Multiply the resulting signal by the ratio of ranges of the “Resegmented signal” and the “Template signal” and normalize the result. This signal, denoted as “Correlation signal”, has local maxima at the start of the intervals along which the “Resegmented signal” and the “Template signal” have the best match, and therefore are more similar in shape and amplitude (figure 2.b). Note that both the “SD Difference signal” and the “Correlation signal” are TL samples shorter than the “Resegmented signal”.

4.5. Calculate the ratio between the “Correlation signal” and the “SD Difference signal” to obtain a new signal, denoted as “Coefficient signal” (figure 2.c).

4.6. Select peaks in the “Coefficient signal” which are found within the dimension of the “Segmented signal” and are located at least a 60% TL samples distant to each other.

4.7. Shift forwards by 15% TL samples the instants at which the peaks were selected, in order to define the instants that approximate heel-strike event in time. The intervals defined between the shifted peaks, named “Events”, allow step durations to be calculated (figure 3.a).

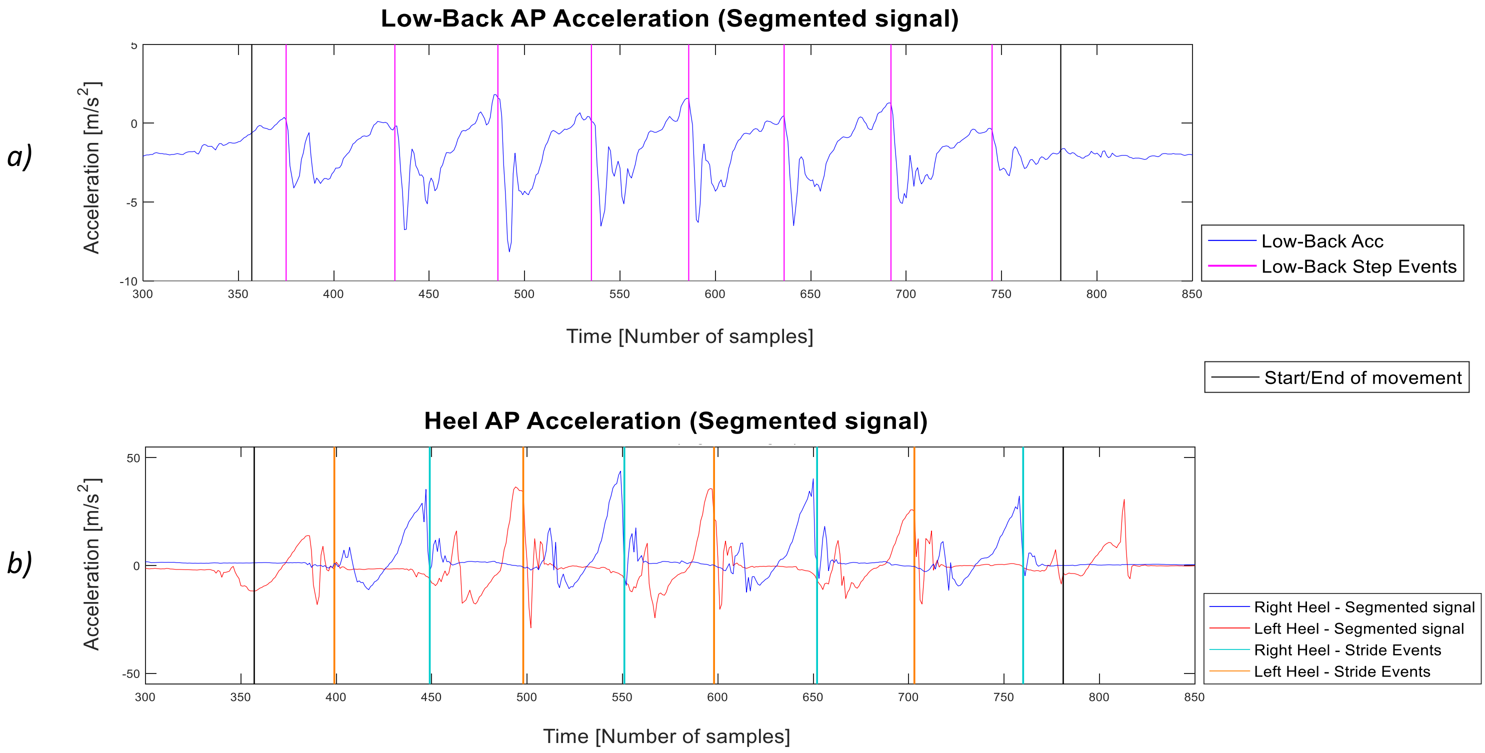

Figure 3. *a. Typical example of a segmented AP acceleration signal collected on the lower back of a patient with PD (blue), and the "Events" (magenta) detected by the algorithm applied to low-back accelerometry.*
b. Typical example of segmented AP acceleration signals collected on the heels of a patient with PD, left heel (red) and right heel (blue), and the respective "Events" detected by the algorithm.

Algorithm for gait segmentation in stride cycles from heel accelerometry

The algorithm applied to the heel accelerations was similar to that of low-back accelerations. However, since heel accelerations have a periodicity in strides instead of steps, the template represents a typical pattern of stride cycle acceleration and it is differently defined. The following steps of the algorithm are different for heel accelerometry.

1.3. The template length (TL) is defined as the resulting number of samples between the first two peaks of the low-pass filtered (cut-off frequency equivalent to the double dominant frequency) normalized unbiased auto-covariance signal which overcome a threshold of 0.5.

2.3. Define sections of the input signal around each peak, starting 5% TL samples before the instant at which the peak is found (instead of 15%, as the slope of the peaks is more steep and TL is about twice long for heel accelerations compared to low-back accelerations) and ending 100% TL samples after the instant at which the peak is found.

In the case of heel accelerometry, the intervals defined between the shifted peaks (5 % TL samples), named “Events”, permit stride durations to be calculated. Thus, left and right stride durations are detected from their respective acceleration signals, and these are combined to obtain step durations (figure 3.b).

Appendix 3

Table 1. Correlation coefficients between significantly different parameters between groups obtained from the self-selected gait speed condition (SS). All the parameters that were not normally distributed and therefore excluded from the discriminant analysis are marked with *, whereas all the selected parameters for the discriminant analysis are marked with a S. The selected parameters are the ones which are normally distributed and independent (non-correlated to any of the selected parameters, i.e. with absolute correlation values lower than 0.7). Absolute correlations above 0.7 are marked in red.

	SELF-SELECTED GAIT SPEED CONDITION (SS)	Duration (Mean across steps)	Duration (Acoustig signal - Start of movement *R)	Duration (Acoustic signal - 1st heel-strike)	Duration (Acoustic signal - 1st heel-strike *R)	Duration (Step Middle)	Duration (Step PreLast)	Displacement (Acoustig signal - Start of movement)	Displacement (Acoustig signal - Start of movement *R)	Displacement (Acoustic signal - 1st heel-strike *R)	Displacement (Step 2 *R)	Displacement (Step Last *R)	Velocity Range AP (Step PreLast)	Velocity Range AP (Step PreLast *R)	Velocity Range AP (Step Last *R)	RMS of Acceleration VT (Acoustig signal - Start of movement)	RMS of Acceleration VT (Acoustig signal - Start of movement *R)	RMS of Acceleration VT (Step 2 *R)	RMS of Acceleration VT (Step PreLast *R)	RMS of Acceleration ML (Acoustig signal - Start of movement *R)	RMS of Acceleration AP (Acoustig signal - Start of movement *R)	RMS of Angular Velocity, around VTL axis (Acoustic signal - Start of movement	RMS of Angular Velocity, around VT axis (Acoustic signal - 1st heel-strike *R)	RMS of Angular Velocity, around ML axis (Acoustic signal - Start of movement	RMS of Angular Velocity, around ML axis (Acoustic signal - 1st heel-strike *R)	RMS of Angular Velocity, around AP axis (Mean across steps)	RMS of Angular Velocity, around AP axis (Acoustic signal - Start of movement	RMS of Angular Velocity, around AP axis (Acoustic signal - 1st heel-strike *R)	RMS of Angular Velocity, around AP axis (Step Middle)	RMS of Angular Velocity, around AP axis (Step PreLast)	RMS of Angular Velocity, around AP axis (Step Last)	RMS of Angular Velocity, around AP axis (Start - End of movement)
	Duration (Mean across steps)	1.00	-0.40	0.13	-0.48	0.91	0.88	-0.36	-0.39	-0.22	0.31	0.09	0.18	0.22	0.03	-0.17	0.25	0.31	0.24	0.21	0.02	0.32	0.19	0.32	0.31	-0.30	0.13	0.19	-0.32	-0.28	-0.26	-0.30
	Duration (Acoustig signal - Start of movement *R)	-0.40	1.00	0.73	0.88	-0.40	-0.32	0.14	0.18	-0.15	-0.22	0.12	0.18	0.04	-0.13	-0.31	-0.58	-0.39	0.13	-0.38	-0.60	-0.31	-0.37	-0.37	-0.37	0.01	-0.40	-0.52	-0.01	0.03	0.02	0.00
	Duration (Acoustic signal - 1st heel-strike)	0.13	0.73	1.00	0.80	0.06	0.15	0.10	0.10	0.19	-0.06	0.27	0.17	0.04	0.03	-0.16	-0.36	-0.21	0.41	-0.35	-0.60	-0.27	-0.35	-0.26	-0.17	0.11	-0.17	-0.22	0.08	0.14	0.19	0.11
S	Duration (Acoustic signal - 1st heel-strike *R)	-0.48	0.88	0.80	1.00	-0.49	-0.39	0.30	0.31	0.33	-0.24	0.16	0.04	-0.09	0.01	-0.02	-0.44	-0.38	0.22	-0.43	-0.52	-0.42	-0.42	-0.37	-0.29	0.30	-0.21	-0.29	0.28	0.31	0.35	0.29
	Duration (Step Middle)	0.91	-0.40	0.06	-0.49	1.00	0.87	-0.37	-0.40	-0.24	0.29	-0.17	0.19	0.19	0.08	-0.11	0.27	0.30	0.14	0.28	0.02	0.25	0.17	0.33	0.32	-0.27	0.21	0.26	-0.32	-0.26	-0.23	-0.26
S	Duration (Step PreLast)	0.88	-0.32	0.15	-0.39	0.87	1.00	-0.37	-0.39	-0.20	0.10	-0.14	0.23	0.30	0.10	-0.04	0.34	0.25	0.18	0.23	0.01	0.27	0.17	0.31	0.34	-0.29	0.22	0.24	-0.33	-0.30	-0.26	-0.28
	Displacement (Acoustig signal - Start of movement)	-0.36	0.14	0.10	0.30	-0.37	-0.37	1.00	0.96	0.54	0.18	0.11	-0.12	-0.23	0.21	0.10	-0.25	-0.03	0.21	-0.37	-0.40	-0.30	-0.21	-0.49	-0.48	0.40	-0.30	-0.18	0.41	0.42	0.37	0.39
S	Displacement (Acoustig signal - Start of movement *R)	-0.39	0.18	0.10	0.31	-0.40	-0.39	0.96	1.00	0.51	0.13	0.07	-0.25	-0.20	0.21	-0.03	-0.29	-0.04	0.11	-0.33	-0.37	-0.20	-0.12	-0.47	-0.49	0.26	-0.35	-0.23	0.28	0.27	0.24	0.25
*	Displacement (Acoustic signal - 1st heel-strike *R)	-0.22	-0.15	0.19	0.33	-0.24	-0.20	0.54	0.51	1.00	0.00	0.07	-0.29	-0.29	0.33	0.51	0.17	0.00	0.20	-0.17	0.03	-0.23	-0.10	-0.11	0.01	0.61	0.24	0.34	0.61	0.58	0.65	0.61
S	Displacement (Step 2 *R)	0.31	-0.22	-0.06	-0.24	0.29	0.10	0.18	0.13	0.00	1.00	-0.16	0.18	0.08	-0.35	0.08	0.28	0.66	-0.06	0.05	0.08	0.32	0.28	0.23	0.23	-0.06	0.16	0.28	-0.10	-0.01	-0.05	-0.05
S	Displacement (Step Last *R)	0.09	0.12	0.27	0.16	-0.17	-0.14	0.11	0.07	0.07	-0.16	1.00	-0.05	-0.09	-0.01	-0.21	-0.46	-0.18	0.46	-0.27	-0.33	-0.28	-0.34	-0.27	-0.21	0.06	-0.33	-0.30	0.15	0.13	0.11	0.05
S	Velocity Range AP (Step PreLast)	0.18	0.18	0.17	0.04	0.19	0.23	-0.12	-0.25	-0.29	0.18	-0.05	1.00	0.51	-0.30	0.15	0.05	0.05	0.13	-0.11	-0.43	-0.07	-0.15	0.00	0.03	0.11	0.12	0.07	0.07	0.12	0.09	0.10
*	Velocity Range AP (Step PreLast *R)	0.22	0.04	0.04	-0.09	0.19	0.30	-0.23	-0.20	-0.29	0.08	-0.09	0.51	1.00	-0.37	-0.11	0.16	0.15	-0.04	0.14	-0.03	0.30	0.11	0.28	0.25	-0.33	0.23	0.21	-0.30	-0.33	-0.33	-0.32
S	Velocity Range AP (Step Last *R)	0.03	-0.13	0.03	0.01	0.08	0.10	0.21	0.21	0.33	-0.35	-0.01	-0.30	-0.37	1.00	0.14	0.00	-0.33	0.24	-0.17	-0.06	-0.32	-0.24	-0.31	-0.33	0.27	-0.10	-0.02	0.27	0.29	0.24	0.27
*	RMS of Acceleration VT (Acoustig signal - Start of movement)	-0.17	-0.31	-0.16	-0.02	-0.11	-0.04	0.10	-0.03	0.51	0.08	-0.21	0.15	-0.11	0.14	1.00	0.70	0.20	0.00	0.01	0.34	-0.07	-0.07	0.12	0.22	0.55	0.71	0.72	0.53	0.51	0.52	0.56
*	RMS of Acceleration VT (Acoustig signal - Start of movement *R)	0.25	-0.58	-0.36	-0.44	0.27	0.34	-0.25	-0.29	0.17	0.28	-0.46	0.05	0.16	0.00	0.70	1.00	0.50	-0.18	0.40	0.69	0.48	0.37	0.50	0.53	0.06	0.79	0.82	0.03	0.04	0.03	0.07
S	RMS of Acceleration VT (Step 2 *R)	0.31	-0.39	-0.21	-0.38	0.30	0.25	-0.03	-0.04	0.00	0.66	-0.18	0.05	0.15	-0.33	0.20	0.50	1.00	-0.26	0.46	0.29	0.54	0.49	0.30	0.37	-0.19	0.39	0.39	-0.21	-0.18	-0.19	-0.18
S	RMS of Acceleration VT (Step PreLast *R)	0.24	0.13	0.41	0.22	0.14	0.18	0.21	0.11	0.20	-0.06	0.46	0.13	-0.04	0.24	0.00	-0.18	-0.26	1.00	-0.40	-0.39	-0.42	-0.52	-0.16	-0.14	0.32	-0.22	-0.12	0.32	0.40	0.37	0.32
S	RMS of Acceleration ML (Acoustig signal - Start of movement *R)	0.21	-0.38	-0.35	-0.43	0.28	0.23	-0.37	-0.33	-0.17	0.05	-0.27	-0.11	0.14	-0.17	0.01	0.40	0.46	-0.40	1.00	0.51	0.66	0.61	0.45	0.48	-0.32	0.31	0.30	-0.33	-0.35	-0.32	-0.32
*	RMS of Acceleration AP (Acoustig signal - Start of movement *R)	0.02	-0.60	-0.60	-0.52	0.02	0.01	-0.40	-0.37	0.03	0.08	-0.33	-0.43	-0.03	-0.06	0.34	0.69	0.29	-0.39	0.51	1.00	0.46	0.43	0.59	0.56	-0.16	0.54	0.56	-0.15	-0.18	-0.18	-0.15
S	RMS of Angular Velocity, around VT axis (Acoustic signal - Start of movement *R)	0.32	-0.31	-0.27	-0.42	0.25	0.27	-0.30	-0.20	-0.23	0.32	-0.28	-0.07	0.30	-0.32	-0.07	0.48	0.54	-0.42	0.66	0.46	1.00	0.89	0.45	0.43	-0.48	0.27	0.29	-0.48	-0.50	-0.44	-0.47
	RMS of Angular Velocity, around VT axis (Acoustic signal - 1st heel-strike *R)	0.19	-0.37	-0.35	-0.42	0.17	0.17	-0.21	-0.12	-0.10	0.28	-0.34	-0.15	0.11	-0.24	-0.07	0.37	0.49	-0.52	0.61	0.43	0.89	1.00	0.35	0.39	-0.38	0.23	0.24	-0.39	-0.43	-0.34	-0.38
*	RMS of Angular Velocity, around ML axis (Acoustic signal - Start of movement *R)	0.32	-0.37	-0.26	-0.37	0.33	0.31	-0.49	-0.47	-0.11	0.23	-0.27	0.00	0.28	-0.31	0.12	0.50	0.30	-0.16	0.45	0.59	0.45	0.35	1.00	0.93	-0.26	0.50	0.48	-0.30	-0.30	-0.25	-0.27
S	RMS of Angular Velocity, around ML axis (Acoustic signal - 1st heel-strike *R)	0.31	-0.37	-0.17	-0.29	0.32	0.34	-0.48	-0.49	0.01	0.23	-0.21	0.03	0.25	-0.33	0.22	0.53	0.37	-0.14	0.48	0.56	0.43	0.39	0.93	1.00	-0.18	0.56	0.53	-0.22	-0.22	-0.14	-0.18
*	RMS of Angular Velocity, around AP axis (Mean across steps)	-0.30	0.01	0.11	0.30	-0.27	-0.29	0.40	0.26	0.61	-0.06	0.06	0.11	-0.33	0.27	0.55	0.06	-0.19	0.32	-0.32	-0.16	-0.48	-0.38	-0.26	-0.18	1.00	0.08	0.09	0.98	0.98	0.96	1.00
*	RMS of Angular Velocity, around AP axis (Acoustic signal - Start of movement *R)	0.13	-0.40	-0.17	-0.21	0.21	0.22	-0.30	-0.35	0.24	0.16	-0.33	0.12	0.23	-0.10	0.71	0.79	0.39	-0.22	0.31	0.54	0.27	0.23	0.50	0.56	0.08	1.00	0.92	0.05	0.05	0.06	0.09
*	RMS of Angular Velocity, around AP axis (Acoustic signal - 1st heel-strike *R)	0.19	-0.52	-0.22	-0.29	0.26	0.24	-0.18	-0.23	0.34	0.28	-0.30	0.07	0.21	-0.02	0.72	0.82	0.39	-0.12	0.30	0.56	0.29	0.24	0.48	0.53	0.09	0.92	1.00	0.05	0.06	0.09	0.11
*	RMS of Angular Velocity, around AP axis (Step Middle)	-0.32	-0.01	0.08	0.28	-0.32	-0.33	0.41	0.28	0.61	-0.10	0.15	0.07	-0.30	0.27	0.53	0.03	-0.21	0.32	-0.33	-0.15	-0.48	-0.39	-0.30	-0.22	0.98	0.05	0.05	1.00	0.96	0.93	0.98
*	RMS of Angular Velocity, around AP axis (Step PreLast)	-0.28	0.03	0.14	0.31	-0.26	-0.30	0.42	0.27	0.58	-0.01	0.13	0.12	-0.33	0.29	0.51	0.04	-0.18	0.40	-0.35	-0.18	-0.50	-0.43	-0.30	-0.22	0.98	0.05	0.06	0.96	1.00	0.94	0.98
*	RMS of Angular Velocity, around AP axis (Step Last)	-0.26	0.02	0.19	0.35	-0.23	-0.26	0.37	0.24	0.65	-0.05	0.11	0.09	-0.33	0.24	0.52	0.03	-0.19	0.37	-0.32	-0.18	-0.44	-0.34	-0.25	-0.14	0.96	0.06	0.09	0.93	0.94	1.00	0.96
*	RMS of Angular Velocity, around AP axis (Start - End of movement)	-0.30	0.00	0.11	0.29	-0.26	-0.28	0.39	0.25	0.61	-0.05	0.05	0.10	-0.32	0.27	0.56	0.07	-0.18	0.32	-0.32	-0.15	-0.47	-0.38	-0.27	-0.18	1.00	0.09	0.11	0.98	0.98	0.96	1.00

Table 2. Correlation coefficients between significantly different parameters between groups obtained from the fast gait speed condition (FS). All the parameters that were not normally distributed and therefore excluded from the discriminant analysis are marked with *, whereas all the selected parameters for the discriminant analysis are marked with a S. The selected parameters are the ones which are normally distributed and independent (non-correlated to any of the selected parameters, i.e. with absolute correlation values lower than 0.7). Absolute correlations above 0.7 are marked in red.

	**FAST GAIT SPEED CONDITION (FS)**	Duration (Mean across steps)	Duration (Step 3)	Duration (Step Middle)	Duration (Step Last)	Displacement (Acoustic signal - Start of movement *R)	Velocity Range AP (Step 2 *R)	RMS of Acceleration VT (Acoustic signal - 1st heel-strike *R)	RMS of Acceleration ML (Step Middle)	RMS of Angular Velocity, around ML axis (Acoustic signal - Start of movement *R)	RMS of Angular Velocity, around ML axis (Acoustic signal - 1st heel-strike *R)	RMS of Angular Velocity, around ML axis (Step PreLast)	RMS of Angular Velocity, around ML axis (Step Last)	RMS of Angular Velocity, around AP axis (Acoustic signal - Start of movement *R)	RMS of Angular Velocity, around AP axis (Step Middle)	RMS of Angular Velocity, around AP axis (Step Middle) *R	RMS of Angular Velocity, around AP axis (Start- End of movement)
	Duration (Mean across steps)	1.00	0.82	0.78	0.47	-0.29	-0.20	0.31	-0.50	0.19	0.27	-0.16	-0.22	0.28	-0.18	-0.06	-0.15
	Duration (Step 3)	0.82	1.00	0.69	0.21	-0.13	-0.13	0.17	-0.36	0.10	0.19	-0.11	-0.05	0.28	-0.08	-0.02	-0.06
S	Duration (Step Middle)	0.78	0.69	1.00	0.23	-0.29	0.12	0.28	-0.24	0.30	0.32	0.03	-0.08	0.30	-0.01	-0.29	0.10
S	Duration (Step Last)	0.47	0.21	0.23	1.00	-0.37	-0.17	0.28	-0.46	0.07	0.15	-0.11	-0.40	0.05	-0.34	-0.07	-0.33
S	Displacement (Acoustic signal - Start of movement *R)	-0.29	-0.13	-0.29	-0.37	1.00	-0.14	-0.08	0.49	-0.45	-0.41	0.35	0.45	-0.17	0.34	0.25	0.23
*	Velocity Range AP (Step 2 *R)	-0.20	-0.13	0.12	-0.17	-0.14	1.00	-0.24	-0.05	0.02	-0.15	0.09	0.05	-0.24	0.11	-0.08	0.13
S	RMS of Acceleration VT (Acoustic signal - 1st heel-strike *R)	0.31	0.17	0.28	0.28	-0.08	-0.24	1.00	-0.40	0.51	0.57	-0.21	-0.45	0.66	-0.21	-0.09	-0.17
S	RMS of Acceleration ML (Step Middle)	-0.50	-0.36	-0.24	-0.46	0.49	-0.05	-0.40	1.00	-0.42	-0.43	0.48	0.57	-0.23	0.50	0.11	0.45
	RMS of Angular Velocity, around ML axis (Acoustic signal - Start of movement *R)	0.19	0.10	0.30	0.07	-0.45	0.02	0.51	-0.42	1.00	0.92	-0.37	-0.52	0.51	-0.31	-0.10	-0.25
S	RMS of Angular Velocity, around ML axis (Acoustic signal - 1st heel-strike *R)	0.27	0.19	0.32	0.15	-0.41	-0.15	0.57	-0.43	0.92	1.00	-0.41	-0.58	0.50	-0.39	-0.14	-0.32
*	RMS of Angular Velocity, around ML axis (Step PreLast)	-0.16	-0.11	0.03	-0.11	0.35	0.09	-0.21	0.48	-0.37	-0.41	1.00	0.76	-0.03	0.77	-0.01	0.78
S	RMS of Angular Velocity, around ML axis (Step Last)	-0.22	-0.05	-0.08	-0.40	0.45	0.05	-0.45	0.57	-0.52	-0.58	0.76	1.00	-0.12	0.66	-0.11	0.69
*	RMS of Angular Velocity, around AP axis (Acoustic signal - Start of movement *R)	0.28	0.28	0.30	0.05	-0.17	-0.24	0.66	-0.23	0.51	0.50	-0.03	-0.12	1.00	-0.13	-0.23	-0.03
*	RMS of Angular Velocity, around AP axis (Step Middle)	-0.18	-0.08	-0.01	-0.34	0.34	0.11	-0.21	0.50	-0.31	-0.39	0.77	0.66	-0.13	1.00	0.19	0.95
S	RMS of Angular Velocity, around AP axis (Step Middle) *R	-0.06	-0.02	-0.29	-0.07	0.25	-0.08	-0.09	0.11	-0.10	-0.14	-0.01	-0.11	-0.23	0.19	1.00	-0.13
*	RMS of Angular Velocity, around AP axis (Start- End of movement)	-0.15	-0.06	0.10	-0.33	0.23	0.13	-0.17	0.45	-0.25	-0.32	0.78	0.69	-0.03	0.95	-0.13	1.00

Appendix 4

Table 3. Mean ± standard deviation values of all calculated kinematic parameters from the self-selected gait speed condition (SS). The top numbers in each cell are the mean and standard deviation values for outcomes from the PDg. The bottom numbers are the mean and standard deviation values for outcomes from the HCg. All parameters that were significantly different between groups are marked with a grey background.

SELF-SELECTED GAIT SPEED CONDITION (SS)	ABSOLUTE									RELATIVE						
	Overall		Initiation of gait		Step-by-step					Initiation of gait		Step-by-step				
	Mean value across steps	Start - End of movement	Acoustig signal - Start of movement	Acoustic signal - 1st heel-strike	Step 2	Step 3	Step Middle	Step Pre-last	Step Last	Acoustic signal - Start of movement *R	Acoustic signal - 1st heel-strike *R	Step 2 *R	Step 3 *R	Step Middle *R	Step Pre-last *R	Step Last *R
Duration [s]	0.59 ± 0.05<br>0.63 ± 0.08	5.24 ± 0.76<br>5.53 ± 0.97	1.18 ± 0.22<br>1.05 ± 0.18	1.45 ± 0.21<br>1.32 ± 0.18	0.62 ± 0.08<br>0.68 ± 0.11	0.58 ± 0.04<br>0.62 ± 0.09	0.55 ± 0.05<br>0.61 ± 0.09	0.58 ± 0.05<br>0.65 ± 0.09	0.65 ± 0.10<br>0.65 ± 0.12	201.92 ± 37.03<br>168.91 ± 33.75	248.62 ± 36.10<br>210.76 ± 32.80	106.19 ± 8.91<br>107.02 ± 8.22	99.22 ± 4.71<br>97.79 ± 6.01	94.11 ± 4.37<br>96.23 ± 5.76	99.87 ± 5.00<br>102.39 ± 5.64	111.24 ± 12.14<br>103.46 ± 10.38
Displacement [m]	0.59 ± 0.07<br>0.60 ± 0.09	4.80 ± 0.07<br>4.84 ± 0.07	0.11 ± 0.03<br>0.07 ± 0.05	0.29 ± 0.16<br>0.23 ± 0.05	0.52 ± 0.09<br>0.57 ± 0.10	0.62 ± 0.09<br>0.63 ± 0.11	0.63 ± 0.07<br>0.66 ± 0.10	0.56 ± 0.09<br>0.56 ± 0.12	0.48 ± 0.10<br>0.43 ± 0.14	18.67 ± 4.39<br>12.42 ± 7.32	48.98 ± 22.92<br>39.44 ± 9.42	89.12 ± 8.72<br>95.32 ± 8.99	104.99 ± 5.83<br>105.24 ± 7.05	108.04 ± 5.57<br>110.74 ± 8.92	95.69 ± 6.63<br>93.66 ± 11.45	80.84 ± 12.41<br>70.59 ± 16.84
Velocity Range AP [m/s]	0.33 ± 0.06<br>0.36 ± 0.08	1.07 ± 0.14<br>1.06 ± 0.17	0.40 ± 0.06<br>0.43 ± 0.08	0.79 ± 0.15<br>0.83 ± 0.15	0.37 ± 0.08<br>0.39 ± 0.13	0.30 ± 0.07<br>0.31 ± 0.09	0.29 ± 0.06<br>0.33 ± 0.09	0.32 ± 0.07<br>0.40 ± 0.09	0.47 ± 0.12<br>0.44 ± 0.11	122.86 ± 17.64<br>121.11 ± 21.61	241.98 ± 49.05<br>238.14 ± 45.06	110.82 ± 16.03<br>108.24 ± 24.88	88.92 ± 14.25<br>86.58 ± 9.09	88.22 ± 12.93<br>91.05 ± 11.53	97.24 ± 13.11<br>111.49 ± 14.12	139.81 ± 19.34<br>123.26 ± 24.86
RMS of Acceleration VT [m/s²]	2.03 ± 0.61<br>1.79 ± 0.58	1.99 ± 0.58<br>1.75 ± 0.56	0.34 ± 0.18<br>0.46 ± 0.20	0.61 ± 0.33<br>0.63 ± 0.20	1.36 ± 0.44<br>1.40 ± 0.42	2.05 ± 0.66<br>1.86 ± 0.75	2.35 ± 0.79<br>2.04 ± 0.75	2.03 ± 0.65<br>1.67 ± 0.61	1.77 ± 0.52<br>1.53 ± 0.48	16.95 ± 5.76<br>26.25 ± 10.33	29.90 ± 9.30<br>36.64 ± 10.85	66.94 ± 8.58<br>79.20 ± 12.37	100.83 ± 9.17<br>100.77 ± 17.46	114.95 ± 8.59<br>113.00 ± 9.29	100.14 ± 8.72<br>92.68 ± 11.93	87.52 ± 8.22<br>86.73 ± 16.08
RMS of Acceleration ML [m/s²]	1.28 ± 0.34<br>1.17 ± 0.34	1.27 ± 0.33<br>1.17 ± 0.35	0.35 ± 0.11<br>0.38 ± 0.07	0.51 ± 0.21<br>0.49 ± 0.08	0.92 ± 0.21<br>0.92 ± 0.25	1.15 ± 0.33<br>1.11 ± 0.44	1.41 ± 0.43<br>1.27 ± 0.45	1.36 ± 0.49<br>1.17 ± 0.33	1.32 ± 0.34<br>1.14 ± 0.29	28.09 ± 7.86<br>34.80 ± 10.32	40.91 ± 11.85<br>44.75 ± 11.19	74.32 ± 14.53<br>80.60 ± 15.72	90.65 ± 10.40<br>93.47 ± 14.45	110.78 ± 16.36<br>107.68 ± 16.12	104.43 ± 15.68<br>101.77 ± 14.11	104.16 ± 12.74<br>98.96 ± 16.08
RMS of Acceleration AP [m/s²]	1.43 ± 0.30<br>1.40 ± 0.44	1.46 ± 0.30<br>1.41 ± 0.42	0.65 ± 0.19<br>0.73 ± 0.14	0.98 ± 0.26<br>1.02 ± 0.18	1.22 ± 0.28<br>1.24 ± 0.39	1.39 ± 0.31<br>1.39 ± 0.42	1.53 ± 0.29<br>1.52 ± 0.49	1.39 ± 0.32<br>1.37 ± 0.50	1.39 ± 0.37<br>1.31 ± 0.47	46.03 ± 11.86<br>54.47 ± 11.48	68.89 ± 13.10<br>75.82 ± 13.33	86.00 ± 8.55<br>88.14 ± 7.56	97.34 ± 5.91<br>99.69 ± 8.57	108.12 ± 10.67<br>108.10 ± 4.56	97.16 ± 9.36<br>97.26 ± 6.67	96.68 ± 11.94<br>92.70 ± 12.84
RMS of Angular Velocity (around VT axis) [deg/s]	0.37 ± 0.19<br>0.32 ± 0.13	0.37 ± 0.19<br>0.32 ± 0.12	0.12 ± 0.04<br>0.14 ± 0.05	0.16 ± 0.08<br>0.16 ± 0.05	0.29 ± 0.13<br>0.29 ± 0.13	0.34 ± 0.18<br>0.31 ± 0.14	0.40 ± 0.23<br>0.34 ± 0.15	0.38 ± 0.26<br>0.32 ± 0.13	0.38 ± 0.20<br>0.31 ± 0.10	34.58 ± 13.91<br>49.37 ± 21.81	44.86 ± 12.09<br>56.45 ± 20.42	80.31 ± 22.02<br>95.70 ± 25.72	91.13 ± 14.95<br>98.39 ± 16.19	109.66 ± 10.97<br>105.14 ± 11.50	99.40 ± 19.64<br>98.81 ± 14.66	106.39 ± 22.86<br>100.74 ± 18.50
RMS of Angular Velocity (around ML axis) [deg/s]	0.28 ± 0.11<br>0.24 ± 0.12	0.28 ± 0.11<br>0.24 ± 0.11	0.11 ± 0.06<br>0.12 ± 0.03	0.13 ± 0.08<br>0.14 ± 0.03	0.23 ± 0.08<br>0.20 ± 0.09	0.28 ± 0.13<br>0.23 ± 0.10	0.31 ± 0.11<br>0.26 ± 0.14	0.29 ± 0.11<br>0.26 ± 0.12	0.26 ± 0.11<br>0.24 ± 0.12	38.28 ± 16.13<br>58.31 ± 26.26	45.31 ± 14.62<br>64.64 ± 26.11	82.21 ± 18.69<br>85.40 ± 22.18	98.24 ± 12.88<br>96.12 ± 19.73	108.72 ± 15.41<br>103.21 ± 13.78	101.36 ± 12.08<br>108.50 ± 22.08	93.78 ± 14.79<br>102.51 ± 26.44
RMS of Angular Velocity (around AP axis) [deg/s]	0.29 ± 0.13<br>0.24 ± 0.11	0.29 ± 0.13<br>0.24 ± 0.11	0.09 ± 0.10<br>0.10 ± 0.05	0.11 ± 0.10<br>0.11 ± 0.05	0.19 ± 0.07<br>0.17 ± 0.09	0.28 ± 0.14<br>0.22 ± 0.11	0.34 ± 0.15<br>0.27 ± 0.12	0.31 ± 0.13<br>0.25 ± 0.11	0.28 ± 0.15<br>0.22 ± 0.13	28.80 ± 11.65<br>45.13 ± 14.79	34.32 ± 12.57<br>48.33 ± 12.90	66.73 ± 15.03<br>70.89 ± 10.79	91.67 ± 10.82<br>93.66 ± 11.27	116.38 ± 10.06<br>112.05 ± 11.21	107.20 ± 8.87<br>105.69 ± 12.18	92.77 ± 13.36<br>90.13 ± 15.29

Table 4. Mean ± standard deviation values of all calculated kinematic parameters from the fast gait speed condition (FS). The top numbers in each cell are the mean and standard deviation values for outcomes from the PDg. The bottom numbers are the mean and standard deviation values for outcomes from the HCg. All parameters that were significantly different between groups are marked with a grey background.

FAST GAIT SPEED CONDITION (FS)	ABSOLUTE									RELATIVE						
	Overall		Initiation of gait		Step-by-step					Initiation of gait		Step-by-step				
	Mean value across steps	Start - End of movement	Acoustig signal - Start of movement	Acoustic signal - 1st heel-strike	Step 2	Step 3	Step Middle	Step Pre-last	Step Last	Acoustic signal - Start of movement *R	Acoustic signal - 1st heel-strike *R	Step 2 *R	Step 3 *R	Step Middle *R	Step Pre-last *R	Step Last *R
Duration [s]	0.48 ± 0.03<br>0.52 ± 0.04	4.05 ± 0.55<br>4.22 ± 0.64	1.04 ± 0.20<br>1.01 ± 0.24	1.23 ± 0.21<br>1.23 ± 0.25	0.51 ± 0.07<br>0.54 ± 0.05	0.49 ± 0.04<br>0.53 ± 0.04	0.47 ± 0.03<br>0.51 ± 0.04	0.51 ± 0.06<br>0.53 ± 0.05	0.46 ± 0.07<br>0.52 ± 0.07	216.28 ± 44.46<br>192.74 ± 44.94	253.90 ± 45.51<br>234.95 ± 45.14	105.36 ± 12.92<br>103.94 ± 6.68	101.13 ± 5.63<br>100.92 ± 4.66	96.15 ± 6.00<br>97.35 ± 3.12	104.50 ± 9.65<br>102.05 ± 7.59	95.81 ± 14.30<br>100.16 ± 13.07
Displacement [m]	0.63 ± 0.08<br>0.67 ± 0.11	4.79 ± 0.06<br>4.81 ± 0.06	0.13 ± 0.04<br>0.11 ± 0.05	0.27 ± 0.07<br>0.28 ± 0.09	0.56 ± 0.13<br>0.63 ± 0.14	0.69 ± 0.12<br>0.74 ± 0.13	0.72 ± 0.11<br>0.75 ± 0.12	0.62 ± 0.12<br>0.65 ± 0.15	0.39 ± 0.14<br>0.45 ± 0.19	20.78 ± 5.18<br>16.89 ± 6.02	43.05 ± 10.58<br>42.56 ± 11.89	87.47 ± 14.09<br>93.57 ± 11.64	109.15 ± 10.08<br>110.83 ± 7.91	113.60 ± 8.46<br>112.69 ± 6.78	97.61 ± 10.43<br>96.25 ± 13.42	60.88 ± 20.96<br>65.32 ± 21.42
Velocity Range AP [m/s]	0.43 ± 0.10<br>0.45 ± 0.09	1.48 ± 0.21<br>1.45 ± 0.21	0.53 ± 0.11<br>0.51 ± 0.08	0.93 ± 0.23<br>0.98 ± 0.20	0.54 ± 0.17<br>0.50 ± 0.13	0.40 ± 0.11<br>0.40 ± 0.08	0.34 ± 0.11<br>0.36 ± 0.09	0.47 ± 0.12<br>0.45 ± 0.14	0.61 ± 0.20<br>0.64 ± 0.17	122.71 ± 19.25<br>116.28 ± 18.51	217.84 ± 52.86<br>221.39 ± 40.44	124.09 ± 30.92<br>110.10 ± 16.74	91.65 ± 17.94<br>90.41 ± 15.00	78.30 ± 15.96<br>79.53 ± 7.72	111.24 ± 28.00<br>101.20 ± 26.12	140.25 ± 33.86<br>144.09 ± 36.73
RMS of Acceleration VT [m/s²]	3.17 ± 0.98<br>2.91 ± 0.78	3.13 ± 0.98<br>2.86 ± 0.77	0.53 ± 0.27<br>0.64 ± 0.33	0.77 ± 0.32<br>0.93 ± 0.40	2.19 ± 0.86<br>2.20 ± 0.60	3.12 ± 1.00<br>3.06 ± 1.01	3.84 ± 1.36<br>3.39 ± 1.06	3.24 ± 1.10<br>2.88 ± 0.90	3.01 ± 1.55<br>2.42 ± 0.89	17.03 ± 6.76<br>21.96 ± 8.43	25.05 ± 9.25<br>32.39 ± 10.19	68.63 ± 13.15<br>76.15 ± 8.63	99.23 ± 17.87<br>103.98 ± 12.97	120.15 ± 10.38<br>115.44 ± 10.21	102.50 ± 16.56<br>98.74 ± 12.94	93.03 ± 26.07<br>82.56 ± 19.07
RMS of Acceleration ML [m/s²]	2.01 ± 0.68<br>1.64 ± 0.51	2.02 ± 0.72<br>1.61 ± 0.49	0.41 ± 0.18<br>0.45 ± 0.15	0.63 ± 0.27<br>0.60 ± 0.15	1.39 ± 0.61<br>1.24 ± 0.39	1.90 ± 0.66<br>1.61 ± 0.61	2.36 ± 0.94<br>1.77 ± 0.67	2.05 ± 0.71<br>1.71 ± 0.71	2.15 ± 1.61<br>1.61 ± 0.55	22.50 ± 11.55<br>29.07 ± 13.03	34.25 ± 17.13<br>39.19 ± 14.77	70.29 ± 21.81<br>77.35 ± 13.62	96.66 ± 22.98<br>99.23 ± 29.05	116.22 ± 25.75<br>106.20 ± 17.46	103.52 ± 20.51<br>103.52 ± 27.23	102.37 ± 33.71<br>99.49 ± 19.29
RMS of Acceleration AP [m/s²]	2.28 ± 0.61<br>2.07 ± 0.54	2.30 ± 0.63<br>2.09 ± 0.54	0.94 ± 0.27<br>0.97 ± 0.28	1.35 ± 0.36<br>1.40 ± 0.46	1.91 ± 0.56<br>1.72 ± 0.34	2.21 ± 0.69<br>1.91 ± 0.45	2.40 ± 0.66<br>2.10 ± 0.64	2.32 ± 0.72<br>2.08 ± 0.68	2.61 ± 1.11<br>2.17 ± 0.87	42.70 ± 11.14<br>48.24 ± 10.81	61.46 ± 15.75<br>68.62 ± 15.12	84.99 ± 17.93<br>85.25 ± 12.23	97.15 ± 15.17<br>93.97 ± 11.97	106.06 ± 13.23<br>101.07 ± 11.97	101.24 ± 13.59<br>100.56 ± 15.96	113.75 ± 31.43<br>103.87 ± 24.73
RMS of Angular Velocity (around VT axis) [deg/s]	0.53 ± 0.28<br>0.44 ± 0.18	0.53 ± 0.27<br>0.44 ± 0.19	0.15 ± 0.07<br>0.15 ± 0.05	0.20 ± 0.08<br>0.18 ± 0.07	0.43 ± 0.25<br>0.37 ± 0.17	0.51 ± 0.30<br>0.45 ± 0.22	0.58 ± 0.31<br>0.46 ± 0.22	0.57 ± 0.34<br>0.47 ± 0.21	0.46 ± 0.22<br>0.43 ± 0.17	30.19 ± 13.51<br>42.17 ± 25.79	39.61 ± 12.42<br>48.99 ± 26.36	82.27 ± 24.90<br>85.09 ± 20.11	98.19 ± 23.22<br>100.48 ± 19.29	108.91 ± 17.01<br>101.53 ± 21.61	106.02 ± 20.55<br>106.31 ± 23.05	90.60 ± 25.17<br>102.38 ± 26.27
RMS of Angular Velocity (around ML axis) [deg/s]	0.43 ± 0.14<br>0.36 ± 0.14	0.42 ± 0.14<br>0.36 ± 0.13	0.16 ± 0.10<br>0.18 ± 0.07	0.18 ± 0.10<br>0.20 ± 0.07	0.33 ± 0.11<br>0.29 ± 0.09	0.43 ± 0.15<br>0.37 ± 0.17	0.48 ± 0.17<br>0.40 ± 0.19	0.46 ± 0.20<br>0.34 ± 0.16	0.45 ± 0.17<br>0.34 ± 0.13	38.92 ± 17.11<br>54.51 ± 23.10	43.02 ± 17.54<br>60.01 ± 20.43	79.68 ± 23.68<br>86.67 ± 22.20	101.34 ± 19.06<br>101.33 ± 20.45	111.38 ± 13.90<br>106.61 ± 19.80	105.91 ± 17.81<br>94.05 ± 16.99	105.77 ± 25.57<br>100.06 ± 33.32
RMS of Angular Velocity (around AP axis) [deg/s]	0.41 ± 0.17<br>0.34 ± 0.12	0.40 ± 0.16<br>0.33 ± 0.11	0.11 ± 0.10<br>0.12 ± 0.05	0.13 ± 0.09<br>0.13 ± 0.05	0.27 ± 0.12<br>0.23 ± 0.09	0.39 ± 0.20<br>0.33 ± 0.13	0.46 ± 0.16<br>0.35 ± 0.12	0.43 ± 0.17<br>0.34 ± 0.12	0.42 ± 0.23<br>0.34 ± 0.18	27.10 ± 8.98<br>36.99 ± 15.10	32.34 ± 14.58<br>39.04 ± 14.64	68.02 ± 21.60<br>68.83 ± 12.34	95.55 ± 17.93<br>97.69 ± 14.42	115.50 ± 15.12<br>104.86 ± 14.93	105.55 ± 19.68<br>101.99 ± 13.77	99.12 ± 23.32<br>97.30 ± 20.36

References of Appendices

1 Rebula J. R., L. V. Ojeda, P. G. Adamczyk and A. D. Kuo. **Measurement of foot placement and its variability with inertial sensors**. *Gait Posture* 38: 974-980, 2013.

2 Srinivasan H., S. Gupta, W. Sheng and H. Chen. **Estimation of hand force from surface Electromyography signals using Artificial Neural Network**. In: *Intelligent Control and Automation (WCICA), 2012 10th World Congress on*IEEE, 2012, p. 584-589.

3 Walgaard S., G. S. Faber, R. C. van Lummel, J. H. van Dieën and I. Kingma. **The validity of assessing temporal events, sub-phases and trunk kinematics of the sit-to-walk movement in older adults using a single inertial sensor**. *Journal of Biomechanics* 2016.
